# Distribution of brain sodium long and short relaxation times and concentrations: a multi-echo ultra-high field ^23^Na MRI study

**DOI:** 10.1038/s41598-018-22711-0

**Published:** 2018-03-12

**Authors:** Ben Ridley, Armin M. Nagel, Mark Bydder, Adil Maarouf, Jan-Patrick Stellmann, Soraya Gherib, Jeremy Verneuil, Patrick Viout, Maxime Guye, Jean-Philippe Ranjeva, Wafaa Zaaraoui

**Affiliations:** 1 Aix-Marseille Univ, CNRS, CRMBM UMR 7339, Marseille, France; 2 APHM, Hôpitaux de la Timone, CEMEREM, Marseille, France; 3 University Hospital Erlangen, Institute of Radiology, Erlangen, Germany; 4 Division of Medical Physics in Radiology, German Cancer Research Centre (DKFZ), Heidelberg, Germany

## Abstract

Sodium (^23^Na) MRI proffers the possibility of novel information for neurological research but also particular challenges. Uncertainty can arise in *in vivo* ^23^Na estimates from signal losses given the rapidity of T2* decay due to biexponential relaxation with both short (T_2_*_short_) and long (T_2_*_long_) components. We build on previous work by characterising the decay curve directly via multi-echo imaging at 7 T in 13 controls with the requisite number, distribution and range to assess the distribution of both *in vivo* T_2_*_short_ and T_2_*_long_ and in variation between grey and white matter, and subregions. By modelling the relationship between signal and reference concentration and applying it to *in vivo* ^23^Na-MRI signal, ^23^Na concentrations and apparent transverse relaxation times of different brain regions were measured for the first time. Relaxation components and concentrations differed substantially between regions of differing tissue composition, suggesting sensitivity of multi-echo ^23^Na-MRI toward features of tissue composition. As such, these results raise the prospect of multi-echo ^23^Na-MRI as an adjunct source of information on biochemical mechanisms in both physiological and pathophysiological states.

## Introduction

In the physiological state, the maintenance of the transmembrane sodium (^23^Na) concentration gradient (10–15 mM intracellular sodium concentration and ~140 mM extracellular sodium concentration) is a precondition for several critical cellular functions. These include the transport of ions, neurotransmitters and nutrients; regulation of osmotic and electrostatic forces on cells and macromolecules; as well as the transmission of action potentials^1–3^. In the diseased state, multiple pathological pathways can lead to aberrations of these functions. Such alterations can lead to changes in intra- and extracellular concentrations due to a reduced ability to maintain resting conditions and due to conformational changes in cells themselves as well as the cellular environment in which they are embedded. Thus, ^23^Na-MRI is a potential source of more direct and quantitative biochemical information than is generally possible with conventional proton (^1^H) MRI^4^. As such, there is substantial interest in ^23^Na-MRI regarding a range of neurological conditions despite the additional complications of acquiring ^23^Na-MRI signal, including stroke^5^, epilepsy^6^, tumors^7^ and neurodegenerative diseases^8–12^.

The quadrupolar ^23^Na nucleus (spin = 3/2) is subject to the influence of fluctuations in neighbouring electric fields due to net positive and non-uniform distribution of charge^3,13–15^. In the presence of a magnetic field, ^23^Na nuclei exhibit four discrete energy states, with three possible (single quantum, SQ) transitions (−1/2, +1/2 “central transition”; −3/2, −1/2 and 1/2, 3/2 “satellite transitions”). In highly motile environments such as plasma or cerebrospinal fluid (CSF), the correlation time (τ_C_) is much shorter than the Larmor period (ω_0_^−1^) (ω_0_∙ τ_C_ ≪ 1), leading to monoexponential longitudinal (T_1_) and transverse (T_2_) relaxation. This is in contrast to tissue environments such as within cells and the interstitial spaces between cells where diffusion is restricted by interactions of the ^23^Na cation with macromolecular anions. Such interactions modulate decay behaviour in a measurable way: the satellite transitions are subject to additional fast relaxation processes so that both T_1_ and T_2_ reflect biexponential decay. Literature T_2_ estimates in brain parenchyma are in the range ~0.5–5 ms and ~15–30 ms for the short and long components of biexponential decay, respectively^2^.

In the environments that give rise to biexponential behaviour, further transitions beyond SQ coherences (SQC) are possible. Such double and triple quantum coherences (D/TQC) can be selectively detected by appropriate multiple quantum filtering (MQF), and thus provide a source of information regarding short and long components of T_2_* decay. However, there is a strong penalty in terms of signal-to-noise ratio (SNR) and resolution for a given acquisition length^2^. Inversion recovery (IR) methods^16–18^ can be used to suppress signal arising from unrestricted aqueous environments such as CSF spaces, though interstitial extracellular areas are not ‘unrestricted’ environments and therefore suppression is incomplete. Furthermore, measurement of concentration remains an unachieved goal for IR methods^3,15^. Both MQF and IR imaging may be argued to provide an unspecified weighting toward intracellular signal beyond the weighted average obtained when measuring the total tissue sodium concentration (TSC). However, all ^23^Na-MRI methods continue to receive contributions from intracellular and extracellular spaces^3,14,15^.

Crucially, the rapid decay of the short component of T_2_ (T_2_*_short_) has direct consequences for the accuracy and SNR of ^23^Na-MRI^19^, leading to signal losses of up to 60%. As such, ^23^Na-MRI acquisition schemes involving ultra-short echo times (UTE, ~0.2 ms) developed as a strategy that minimises uncertainty in T_2_ signal loss due to biexponential relaxation^20,21^. However, this does not address variation in T_2_ that may be influenced by methodological or physiological factors. The latter in particular may be of interest in the interpretation of ^23^Na-MRI signal as a measure of healthy and aberrant biochemical function. Accurate *in vivo* multi-echo relaxometry techniques may provide an adjunct source of information regarding the contribution and distribution of T_2_*_short_ and T_2_*_long_. Previous attempts to characterise *in vivo* brain T_2_ decay times have been conducted at lower field strengths with lower sampling rates in the temporal domain without separating multiple T_2_* components^22–24^, or at higher fields only provide estimates of T_2_*_long_^25,26^ or did not consider variation between tissue types (white matter, WM; grey matter, GM)^18^ or between different regions^27,28^.

**Figure 1:**
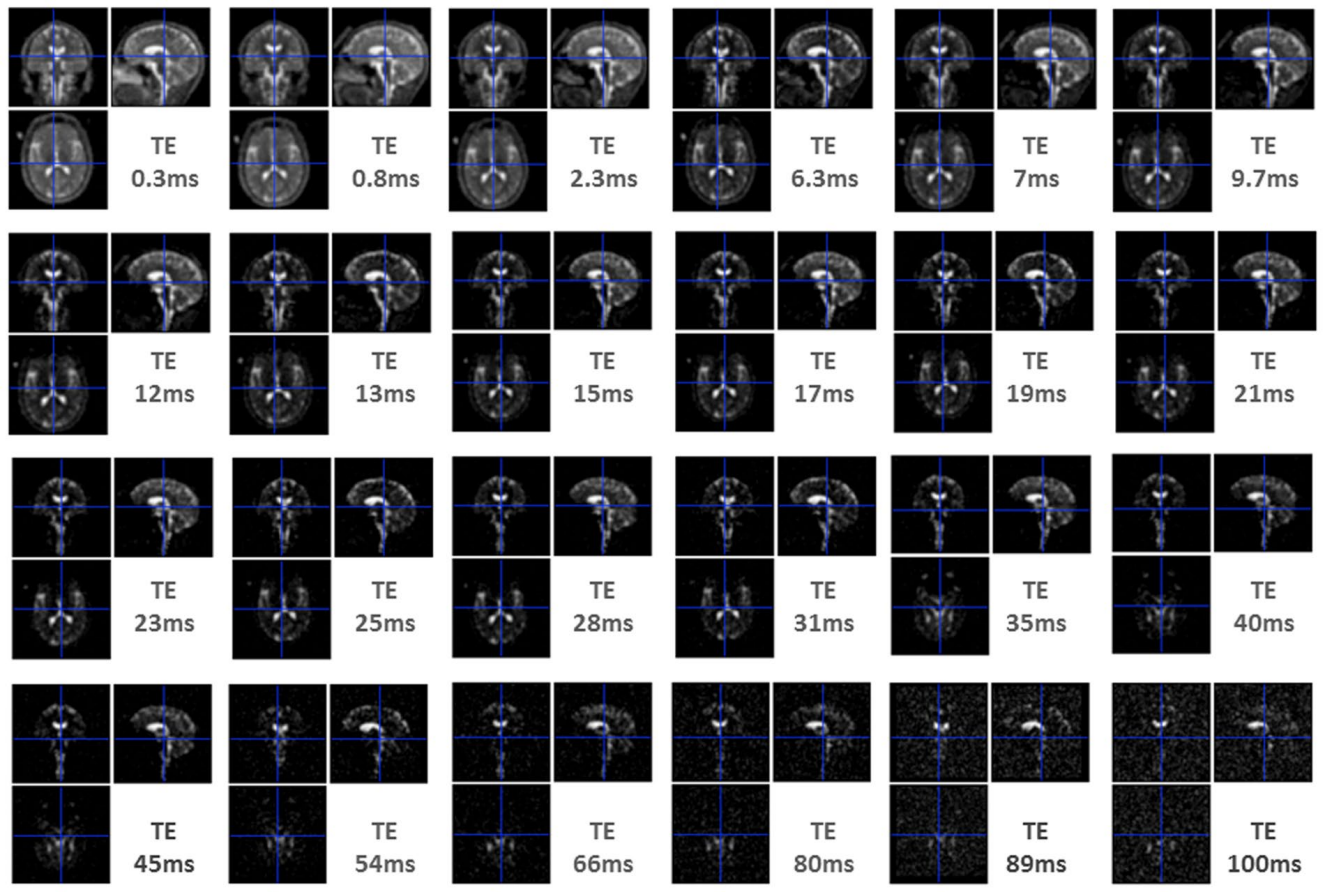
Exemplar ^23^Na-MRI images from a single subject across echo times. Note the decrease in signal as a function of the length of echo time (TE), and the change in the relative contributions from tissue and cerebrospinal fluid, with the latter providing most signal at long TEs.

**Figure 2:**
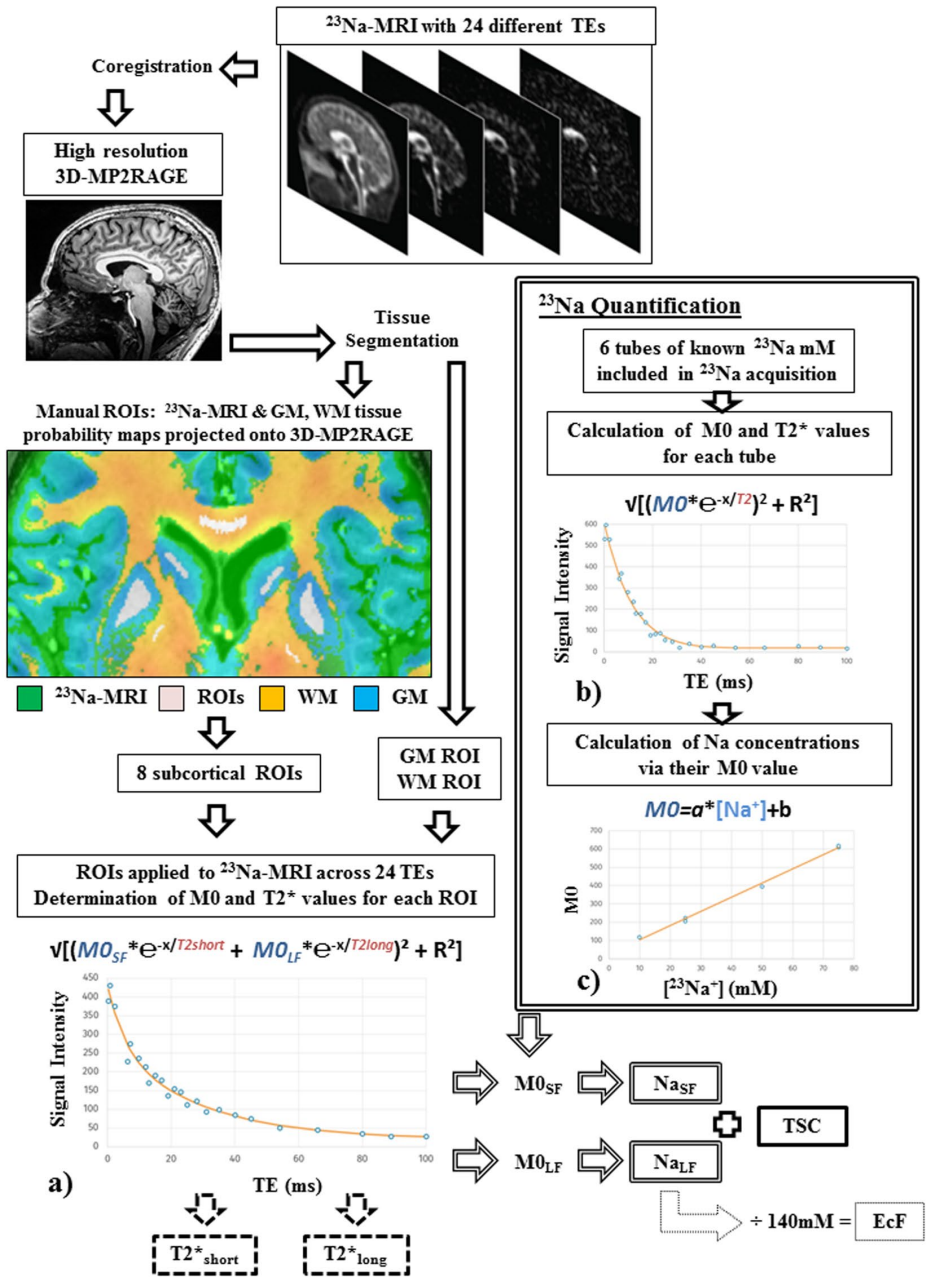
Schematic workflow of processing procedure applied to data and the six measures obtained. After image post-processing, several fitting procedures were applied to ^23^Na-MRI data derived from 10 ROIs (examples shown include the caudate, putamen, pallidum and corpus callosum). Fitting included: (**a**) a biexponential model to obtain estimates of the magnetization (M0_SF_, M0_LF_) apparent from the signal fractions associated with short and long T_2_* components from *in vivo* ROIs; (**b**) a monoexponential model to obtain estimates of M0 for each reference tube and (**c**) a linear model of the relationship between M0 and known reference concentrations. The six output parameters and their derivation are marked: two T_2_* components (dashed outline), a Na concentration for each component (double outline), TSC (solid outline) and EcF (dotted outline) See text for more details. *Abbreviations*: TE, echo time; mM, millimolar; ms, milliseconds; ROIs, regions of interest.

Here, we designed a novel approach based on the density adapted radial sequence^29^, at 7 T with multiple-echoes (n = 24) (Fig. 1) ensuring echo time (TE) sampling with the requisite number, distribution and range to robustly assess the distribution of both *in vivo* T_2_*_short_ and T_2_*_long_ through biexponential modelling. Modelling was applied to empirical estimates of sodium signal in healthy volunteers between WM and GM and, for the first time, among several subcortical regions. In addition the fraction of signal or magnetization (M0) at short TEs (M0_SF_) and at long TEs (M0_LF_) was converted to concentrations, by modelling the relationship between ^23^Na signal (M0) and concentration (mM) in reference tubes. As such, *in vivo* ^23^Na concentrations (Na_SF_, Na_LF_) corresponding to different signal fractions for the various tissues and regions were provided for the first time (Fig. 2).

## Results

Figure 2 provides an overview of the workflow applied to data, including the extraction of decay time components (T_2_*_short_ and T_2_*_long_), concentrations (Na_SF_, Na_LF_), total sodium concentration (TSC) and extracellular fraction (EcF). See Table 1 for mean estimates of each parameter for each tissue type and region.

**Table 1:** Mean (*Mn*) and standard deviation (*s*.*d*) for each dependant variable for each tissue type and region. EcF, extracellular; GM, grey matter; TSC, total sodium concentration. WM, white matter.

		T_2_*_short_ (ms)	T_2_*_long_ (ms)	Na_SF_ (mM)	Na_LF_ (mM)	TSC (mM)	EcF
GM	***Mn***	**4.99**	**31.51**	**21.16**	**25.75**	**46.92**	**0.18**
	*s*.*d*	*0.41*	*1.78*	*0.87*	*2.97*	*2.83*	*0.02*
WM	***Mn***	**4.44**	**38.25**	**22.28**	**15.87**	**38.15**	**0.11**
	*s*.*d*	*0.34*	*2.45*	*0.97*	*1.49*	*1.97*	*0.01*
Thalamus	***Mn***	**3.70**	**40.13**	**24.28**	**16.02**	**40.30**	**0.11**
	*s*.*d*	*0.41*	*7.50*	*1.66*	*2.85*	*2.33*	*0.02*
Putamen	***Mn***	**3.72**	**32.66**	**23.04**	**13.25**	**36.29**	**0.09**
	*s*.*d*	*0.61*	*7.00*	*2.82*	*3.19*	*1.76*	*0.02*
Pallidum	***Mn***	**2.82**	**31.62**	**21.99**	**11.25**	**33.24**	**0.08**
	*s*.*d*	*0.44*	*6.87*	*2.08*	*2.62*	*2.62*	*0.02*
Caudate	***Mn***	**4.00**	**34.79**	**19.26**	**17.80**	**37.06**	**0.13**
	*s*.*d*	*0.61*	*6.37*	*2.10*	*2.95*	*2.09*	*0.02*
Corpus Callosum	***Mn***	**3.48**	**39.82**	**19.57**	**15.96**	**35.52**	**0.11**
	*s*.*d*	*0.42*	*9.16*	*3.30*	*4.87*	*3.35*	*0.03*
Cerebellar WM	***Mn***	**3.57**	**35.51**	**16.29**	**12.31**	**28.60**	**0.09**
	*s*.*d*	*0.81*	*7.26*	*2.34*	*2.35*	*2.88*	*0.02*
Centrum Semiovale	***Mn***	**4.37**	**54.74**	**23.93**	**8.46**	**32.39**	**0.06**
	*s*.*d*	*0.43*	*14.98*	*2.83*	*1.68*	*2.74*	*0.01*
Pons	***Mn***	**2.43**	**31.52**	**13.19**	**11.99**	**25.18**	**0.09**
	*s*.*d*	*0.58*	*11.45*	*3.68*	*3.53*	*3.17*	*0.03*

**Figure 3:**
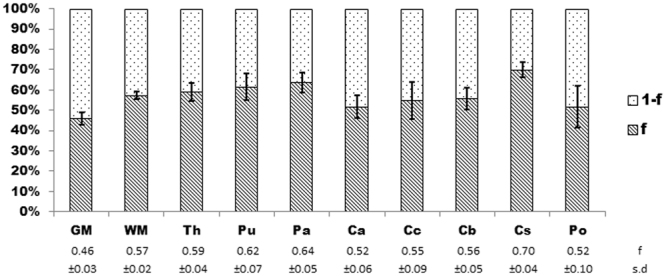
Estimated percentage signal contributions of short (f) and long (1-f) T_2_* components. Mean and standard deviation (s.d) for f are provided at the bottom of the figure, and s.d. is represented as error bars on the stacked chart. Long component percentage contributions are necessarily the inverse (1-f). ROIs: Th, thalamus; Pu, putamen; Pa, pallidum; Ca, caudate; Cc, corpus callosum; Cb, cerebellum, Cs, centrum semiovale; Po, pons.

Goodness of fit indicated high performance of the biexponential decay model across the 10 ROIs including complete GM and WM segmentations and eight sub-cortical ROIs (mean r^2^ = 0.97 ± 0.01(s.d.)). The mean proportion of the signal fraction (*f*) associated with the short T_2_* component to total signal estimated by the biexponential model is 57.7% ± 0.07 (s.d.). There was also variation among ROIs (Fig. 3) with the overall highest value obtained for the centrum semiovale (70% ± 0.04 (s.d.)). The caudate nucleus (52% ± 0.06 (s.d.)) and pons (52% ± 0.1 (s.d.)) jointly provide the lowest estimates among subcortical regions, while the lowest mean estimate across all ROIs was for the entire GM segmentation (46% ± 0.03(s.d.)).

### T_2_*_short_ and T_2_*_long_ differences between white and grey matter

Table 2a provides results for the whole model (ANOVA, corrected at *P* < 0.008) and main effects of Tissue Type (GM, WM) for each parameter. Effects of Age and Sex were not found to be significant for any parameter. Significant effects were observed for whole model for T_2_*_long_ (*P* < 0.0001) and T_2_*_short_ (*P* = 0.005) as well significant effects of tissue type for T_2_*_long_ (*P* < 0.0001) and T_2_*_short_ (*P* = 0.001).

**Table 2:** Significant (*P* < 0.008) main effects (F). Results of ANOVAs applied to a) data from whole GM, WM masks and b) sub-regions. Numbers in brackets indicate degree of freedom. Non-significant main effects indicated by a dash (—). EcF; extracellular fraction; TSC, total sodium concentration.

		F-Values			
	Dependant variable	Whole Model (3)	Tissue Type (1)	Sex (1)	Age (1)
**a)**	T_2_*_short_	5.75	14.39	—	—
	T_2_*_long_	26.07	72.95	—	—
	Na_SF_	—	—	—	—
	Na_LF_	36.7	109.63	—	—
	TSC	29.25	85.33	—	—
	EcF	36.7	109.31	—	—
	**Dependant variable**	**Whole Model (9)**	**Region (7)**	**Sex (1)**	**Age (1)**
**b)**	T_2_*_short_	12.44	15.97	—	—
	T_2_*_long_	7.23	9	—	—
	Na_SF_	24.41	30.02	—	—
	Na_LF_	9.5	12.13	—	—
	TSC	37.36	46.64	8.03	—
	EcF	9.5	12.13	—	—

**Figure 4:**
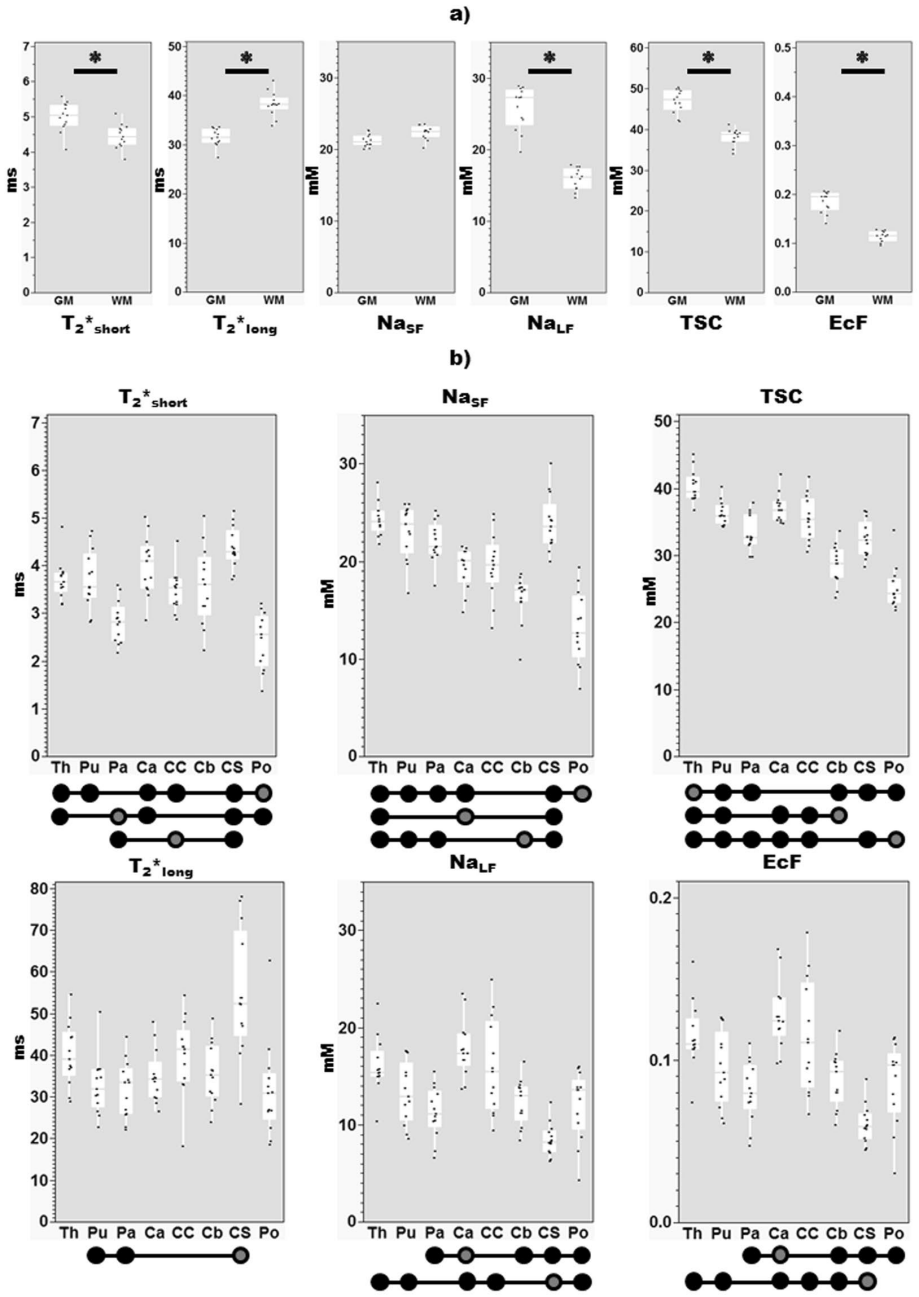
Post-hoc differences (z, Steel-dwass). Note the y-axis scale differs between parameters. Results of tests applied to (**a**) data from whole GM, WM masks (Significant differences of *P* < 0.008 marked with a bar and asterisk), and (**b**) sub-regions. Due to the number of comparisons between subregions, significant differences at Bonferroni-corrected level between a region (circle with dark grey interior) with other regions (full black circles) are indicated below each graph. *Abbreviations:* EcF, extracellular fraction; GM, grey matter; mM, millimolar; ms, milliseconds; TSC, total sodium concentrations; WM, white matter; Th, thalamus; Pu, putamen; Pa, pallidum; Ca, caudate; Cc, corpus callosum; Cb, cerebellar WM, Cs, centrum semiovale; Po, pons.

Results of post-hoc Steel-Dwass tests are detailed in Table 3 and represented in Fig. 4. Significantly higher (z = −3.03, *P* < 0.003) values of T_2_*_short_ were observed in GM (4.99 ms ± 0.41(s.d.)) versus WM (4.44 ms ± 0.34(s.d.). Conversely, higher T_2_*_long_ estimates were observed in WM (38.25 ms ± 2.45 (s.d.)) versus GM (31.51 ms ± 1.78(s.d.)) (z = 4.31, *P* < 0.0001).

**Table 3:** Significant differences between regions. Differences (z) are provided for each significant comparison (*P* < 0.008), with the corresponding *P* value provided in italics, and non-significant comparisons indicated by a dash (—). Diagonal elements with asterisks represent borders between the results of different tests, for example in the top row the bottom triangle represents results between regions for T_2_*_short_, while the upper triangle represents results between regions for T_2_*_long_.  Th, thalamus; Pu, putamen; Pa, pallidum; Ca, caudate; Cc, corpus callosum; Cb, cerebellar WM, Cs, centrum semiovale; Po, pons.

		Th	Pu	Pa	Ca	Cc	Cb	Cs	Po		
T_2_*_short_	Th	********	—	—	—	—	—	—	—	Th	T_2_*_long_
	Pu	—	********	—	—	—	—	—3.59,0.008	—	Pu	
	Pa	3.79,0.004	—	********	—	—	—	−3.79,0.004	—	Pa	
	Ca	—	—	−3.74,0.005	********	—	—	—	—	Ca	
	Cc	—	—	—	—	********	—	—	—	Cc	
	Cb	—	—	—	—	—	********	—	—	Cb	
	Cs	—	—	−4.31,0.0004	—	3.74,0.005	—	********	—	Cs	
	Po	4.21,0.0007	3.9,0.003	—	−4.15,0.0009	−3.95,0.002	—	−4.31,0.0004	********	Po	
		**Th**	**Pu**	**Pa**	**Ca**	**Cc**	**Cb**	**Cs**	**Po**		
Na_SF_	Th	********	—	—	—	—	—	4.21,0.0007	—	Th	Na_LF_
	Pu	—	********	—	—	—	—	3.64,0.007	—	Pu	
	Pa	—	—	********	—3.95,0.002	—	—	—	—	Pa	
	Ca	4.31,0.0004	—	—	********	—	—3.79,0.004	—4.3,0.0004	—3.74,0.005	Ca	
	Cc	—	—	—	—	********	—	—4,0.002	—	Cc	
	Cb	4.31,0.0004	3.9,0.003	4.15,0.0009	—	—	********	—	—	Cb	
	Cs	—	—	—	3.85,0.003	—	4.31,0.0004	********	—3.64,0.007	Cs	
	Po	4.31,0.0004	4.15,0.0009	—4.21,0.0007	—3.69,0.006	—	—	−4.31,0.0004	********	Po	
		**Th**	**Pu**	**Pa**	**Ca**	**Cc**	**Cb**	**Cs**	**Po**		
TSC	Th	********	—	—	—	—	—	4.21,0.0007	—	Th	EcF
	Pu	3.74,0.005	********	—	—	—	—	3.64,0.007	—	Pu	
	Pa	4.25,0.0005	—	********	−3.95,0.002	—	—	—	—	Pa	
	Ca	—	—	—	********	—	−3.79,0.004	−4.3,0.0004	−3.74,0.005	Ca	
	Cc	—	—	—	—	********	—	−4, 0.002	—	Cc	
	Cb	4.31,0.0004	4.31,0.0004	—	−4.31,0.0004	3.9, 0.003	********	−3.64,0.007	—	Cb	
	Cs	4.31,0.0004	—	—	—	—	—	********	—	Cs	
	Po	4.31,0.0004	4.31,0.0004	−3.85,0.003	−4.31,0.0004	−4.1,0.001	—	−3.85,0.003	********	Po	
		**Th**	**Pu**	**Pa**	**Ca**	**Cc**	**Cb**	**Cs**	**Po**

### T_2_*_short_ and T_2_*_long_ differences between sub-cortical regions

ANOVAs revealed significant (*P* < 0.0001) effects for both the whole model and the effect of Region (Table 2b) on both T_2_*_short_ and T_2_*_long_. Overall, in terms of the post-hoc analysis (Table 3, Fig. 4b), we observed substantial variation for each parameter between subcortical regions. The shortest mean decay time components were observed in the pons for both T_2_*_short_ (2.43 ms ± 0.58(s.d.)) and T_2_*_long_ (31.52 ms ± 11.45(s.d.)), with the longest observed in the centrum semiovale (T_2_*_short_: 4.37 ms ± 0.43(s.d.); T_2_*_long_: 54.74 ms ± 14.98(s.d.)). The centrum semiovale also evidenced a wide distribution of T_2_*_long_ estimates between subjects.

### Concentration differences between white and grey matter

The whole model and the main effect of tissue type were significant (*P* < 0.0001) for Na_LF_, TSC and EcF. Whole model and tissue type effects for Na_SF_ did not survive correction for multiple comparisons. Post-hoc Steel-Dwass tests revealed significantly higher values in GM versus WM for Na_LF_ (25.75 mM ± 2.97(s.d.), 15.87 mM ± 1.49(s.d.); z = −4.31, *P* < 0.0001), extracellular fraction (0.18 ± 0.02(s.d.), 0.11 ± 0.01(s.d.); z = −4.31, *P* < 0.0001) and TSC (46.92 mM ± 2.83(s.d.), 38.15 mM ± 1.97(s.d.); z = −4.31, *P* < 0.0001).

### Concentration differences between sub-cortical regions

Both NaSF and NaLF, as well as TSC and EcF, demonstrated significant (*P* < 0.0001) effects for both the whole model and the effect of Region. A significant effect of Sex was also observed for TSC (*P = *0.006). As above, concentrations and derived metrics showed a variety of variations between regions. The thalamus demonstrated the highest mean estimates of Na_SF_ (24.28 mM ± 1.66(s.d.)) and TSC (40.30 mM ± 2.33(s.d.)), with the lowest estimates observed in the pons (Na_SF_: 13.19 mM ± 3.68(s.d.); TSC: 25.18 mM ± 3.17(s.d.)). The caudate (Na_LF_: 17.80 mM ± 2.95(s.d.); EcF: 0.13 ± 0.02(s.d.)) and centrum semiovale (Na_LF_: 8.46 mM ± 1.68(s.d.); EcF: 0.06 ± 0.01 (s.d.)), provided the highest and lowest estimates, respectively, for both Na_LF_ and EcF. Inter-subject variability was especially present for EcF and Na_LF_ in the corpus callosum.

## Discussion

Data from a multi-echo ^23^Na radial sequence acquired at ultra-high field (UHF) were analysed using a biexponential relaxation model to derive T_2_*_short_ and T_2_*_long_ relaxation times. In addition, ^23^Na concentrations apparent at short and long TEs were quantified. As such, to our knowledge, we provide the first ^23^Na-MRI data to address regional variation in T_2_*_short_ at UHF, and to quantify the apparent ^23^Na concentrations corresponding to short and long signal fractions associated with the respective components of T2* relaxation. Given the interest in ^23^Na-MRI particularly for neurological research, quantification of these parameters and characterisation of their variation is necessary as an adjunct to even standard UTE ^23^Na-MRI protocols.

The overall contribution of short and long T_2_* components to the measured sodium signal is considered to be ~60% to 40%^2^, respectively, consistent with our mean estimate of 57.7% ± 0.07% (s.d.) for the signal fraction associated with the short T_2_* component (*f*) across all considered ROIs. In the biexponential relaxation model applied here contributions of different T_2_* components were not fixed. The model revealed that while *f* was comparable to the general estimate in some regions (eg the entire WM), it varied in others (eg. the lowest signal contributions over the entire GM) (Fig. 3). Additionally, variation was observed across sub-cortical ROIs which are themselves likely to include diverse proportions of grey and white matter. Examples include regions that mainly contain white matter tracts but also grey matter nuclei (eg. Pons), as well as the inverse (eg. thalamus). Deviation from the theoretical ratio of 60:40 may reflect the contribution of additional compartments, for example an increased contribution of highly motile ions pool in CSF where signal decay is monoexponential (eg T_2_* ~ 47 ms)^27^. Higher estimates of *f* may reflect reduced contributions of this type, for example in the centrum semiovale which provided the highest signal fraction associated with the short T_2_* component observed here.

T_2_* relaxation refers to the dephasing of collections of nuclei due to reciprocal interactions of their local magnetic fields (T_2_) in addition to magnetic field inhomogeneities (T_2_’)^30^. Attempts to characterize *in vivo* ^23^Na relaxation times in human brain tissue have been made for several decades^22,24^. Such applications show variation in terms of applied methods, following developments in sequence design and the availability of higher field strengths. These factors are especially important for ^23^Na, given not only the relative rapidity of biexponential relaxation but also due to its lower concentration and intrinsic sensitivity compared to ^1^H. This leads to a relatively lower SNR in ^23^Na-MRI, which can be partly compensated for by higher fields^3,31^. Consequently, among studies that attempt to quantify either long or short components of T_2_* relaxation in brain tissues (for an overview see Table 4), progressively higher fields have been employed. At 4 T, values of T_2_*_long_ were observed to vary little between cortical GM and WM ROIs, though with some evidence of variation in subcortical regions (thalamus) and higher values in some white matter structures like the corpus callosum (CC)^25^. In line with theoretical expectations, and as observed in animal experiments^32^, longer relaxation times are observed at the higher field strengths in the current data (Table 1) and more generally at 7 T (Table 4). At 7 T, despite the lack of variation in T_2_*_long_ values between general GM and WM ROIs, variation among other sub-regions comparable to the relative levels of T_2_*_long_ seen in the current work was observed (thalamus > CC > cerebellar WM > putamen)^26^. Due to insufficient data points to characterize the initial portion of the decay curve, given that the smallest TEs are > 3 ms, these studies did not attempt to quantify T_2_*_short_. Thus the possibility of an unknown bias in measurement, relative to studies that model both T_2_* components remains. In a 7 T study in healthy controls of both T_2_*_short_ and T_2_*_long_ in white matter, estimates in excellent agreement with the current data were observed by Nagel *et al*.^18^. More recent data^27^ in five subjects provide similarly concordant data for GM, though some disagreement for relative T_2_*_short_ relaxation times between tissues should be noted. Recently, Blunck *et al*.^28^ validated a multi-echo sequence with a large numbers of TEs, estimating decay components in 4 subjects, and observe T_2_*_short_ relaxation times that are within the range of those seen here, as well as less variation between GM and WM for T_2_*_long_. The principal methodological differences between this study and the previous studies characterising both T_2_*_short_ and T_2_*_long_ in GM and WM at 7 T, beyond a slightly larger sample, is the distribution of early TEs during the first part of the decay curve thought to be dominated by the short T_2_* component. For example the three shortest TEs correspond to 0.3–0.8–2.3 ms here, versus 0.4–4.5–8.5 ms for Blunck *et al*.^28^ and 0.35–1–1.5 ms for Niesporek *et al*.^27^. The echo time choices made here are dictated by the minimum inter-echo spacing of about 6 ms. With 3 separate acquisitions there is additional flexibility in the TE choices. The approach adopted was to use logarithmic spacing for the first echoes and equal spacing for later echoes. Logarithmic spacing on the early echoes provides good sensitivity to a range of T_2_*_short_ values. The current data suggest that GM and WM give rise to significantly different time constants of decay, and do so differentially for short and long T_2_* components. These considerations, in addition to the variation among subregions and between T_2_* components for given regions, raise the possibility of the influence of tissue composition on observed relaxation (discussed below).

**Table 4:** Examples of literature estimates for short and long component of T_2_* derived using biexponential models, and CSF transverse relaxation rates using monoexponential models. Note that data is attributed to structures as described in the papers concerned, and the details of ROIs likely differ between reports. Brain parenchyma refers to estimates derived from (an) ROI(s) where no specific anatomic attribution was made. Values reproduced from Bartha and Menon^25^, Fleysher *et al*.^26^, Nagel *et al*.^18^, Blunck *et al*.^28^ and Niesporek *et al*.^27^. *Abbreviations:* Ref, reference; T, Tesla; TE, echo time; GM, grey matter; WM, white matter.

Structure	Ref	Field Strength (T)	TE n:range (ms)	T_2_*_short_ (ms)		T_2_*_long_ (ms)	
				GM	WM	GM	WM
Brain Parenchyma	^25^	4	10: 3.8–68.7	—	—	21.1 ± 0.5	
	^26^	7	2: 12–32	—	—	28 ± 2	29 ± 2
	^18^	7	56: 0.35–55	—	4.7 ± 0.2	—	40 ± 2
	^28^	7	38: 0.4–150	2.02 ± 1.67	1.99 ± 2.09	25.9 ± 8.3	22.4 ± 7.8
	^27^	7	32: 0.35–85	5.4 ± 0.2	3.5 ± 0.1	36.4 ± 3.1	23.3 ± 2.6
Frontal lobe	^26^	7	2: 12–32	—	—	27 ± 2	31 ± 1
Prefrontal	^25^	4	10: 3.8–68.7	—	—	21.2 ± 2.2	19.3 ± 2.9
Occipital Lobe	^26^	7	2: 12–32	—	—	29 ± 1	28 ± 1
	^25^	4	10: 3.8–68.7	—	—	22 ± 2.4	—
Parietal lobe	^25^	4	10: 3.8–68.7	—	—	—	18.2 ± 1.9
Insula	^25^	4	10: 3.8–68.7	—	—	21.6 ± 1.2	—
External capsule	^25^	4	10: 3.8–68.7	—	—	—	18.3 ± 2.9
Thalamus	^26^	7	2: 12–32	—	—	32 ± 2	—
	^25^	4	10: 3.8–68.7	—	—	16.9 ± 2.4	—
Putamen	^26^	7	2: 12–32	—	—	23 ± 2	—
Peri-ventricle WM	^26^	7	2: 12–32	—	—	—	34 ± 1
Cerebellum	^26^	7	2: 12–32	—	—	—	24 ± 1
Splenium of CC	^26^	7	2: 12–32	—	—	—	28 ± 2
CC	^25^	4	10: 3.8–68.7	—	—	—	22 ± 2.7
CSF	^26^	7	2: 12–32	54 ± 4			
	^25^	4	10: 3.8–68.7	63.7 ± 4.7			
	^28^	7	38: 0.4–150	57.2 ± 6.6			
	^18^	7	56: 0.35–55	56 ± 4			
	^27^	7	32: 0.35–85	46.9 ± 2.1			

By modelling the relationship between signal (M0) and known reference concentrations, we obtained – from estimates of M0_SF_ and M0_LF_ derived from *in vivo* data – quantitative values for the apparent ^23^Na (Na_SF_, Na_LF_) concentrations relating to short (*f*) and long (1-*f*) signal fractions (Fig. 2). Na_SF_ was observed to be comparable between GM (21.16 mM ± 0.87(s.d.)) and WM (22.28 mM ± 0.97(s.d.)). These values are higher than the reference intracellular sodium concentration (10–15 mM) commonly cited in the literature^1–3^, possibly reflecting contributions from extracellular spaces. Various attempts have been made to weight measures of concentration toward the restricted sodium pool. For example, subtraction of only two SQ sodium images as a means of suppressing contributions from the long T_2_* component obtains a concentration of 25.9 ± 1.2 mM for bound sodium in the ‘healthy’ GM of patients with brain tumours^33^. This estimate is not dissimilar to our estimates of GM though most similar to the Na_LF_ concentration we observe in WM (Table 1). Recent studies – utilizing IR at 3T^4,34^ and TQF at UHF^10,35^ – find little difference in concentration estimates weighted toward the restricted pool between GM and WM in their healthy control comparison samples. This is consistent with the similarity between our estimates of Na_SF_ from whole tissue type segmentation maps for GM (21.16 mM ± 0.87(s.d.) and WM (22.28 mM ± 0.97(s.d.)) (Fig. 4a). Similarly, attempts to measure volume fractions associated with bound sodium (WM > GM)^10,35^, are complementary with the current work’s estimate of sodium extracellular fraction (GM > WM) derived from Na_LF_. Indeed, the sodium EcF estimates observed here (0.06–0.18) fall well within the expected overall volume fraction of the extracellular space in healthy adult brains (0.2) derived from a variety of methods^36^. Furthermore, they specifically correspond to previous estimates of greater ^23^Na extracellular volume fractions in grey versus white matter using ^23^Na-MRI^4,34^.

Most *in vivo* studies of brain ^23^Na-MRI provide estimates in terms of total sodium concentration (TSC), thus we computed an equivalent metric to permit comparison. Estimates here (GM: 46.92 mM ± 2.83 (s.d.); WM: 38.15 mM ± 1.97 (s.d.)) fall well within the literature range for TSC in GM (30–70 mM) and WM (20–60 mM)^2,14^. Specifically, in the majority of studies that report healthy control values in GM, TSC is observed to exhibit higher values compared to WM, including across field strengths and different acquisitions^8,10,12,37–40^. Niesporek *et al*.^41^, employing a partial volume correction method adapted from PET accounting for both point spread function spreading and resolution issues in ^23^Na-MRI, suggest corrected TSC values for GM (48 mM ± 1 (s.d.)) and WM (43 mM ± 3(s.d.)) notably similar to our estimates. Where available, regional TSC estimates in healthy controls are concordant with the current results in terms of finding higher values in the thalamus and caudate compared to putamen and pallidum as well as much lower values in the cerebellum^11^, and specifically its white matter^37^. The correspondence of our observed TSC values (the sum of Na_SF_ and Na_LF_) and those in the literature are encouraging, suggesting that the current methods appropriately characterize the overall ^23^Na signal with respect to the rest of the literature. This lends further credence to the signal fractions and concentrations associated with the short and long T_2_* components as observed here. Crucially, the current methods achieve this without a high SNR penalty and permit quantification.

Relaxation is influenced by factors constraining molecular motion as well as the structure and content (tissue density) of the molecular environment experienced by nuclei^30^. For example, one factor of tissue density is water/fluid content which can be explored via invasive electrophysiological measures of impedance^42^, *in vivo* MRI estimates of proton density^43^ and post-mortem wet-to-dry mass ratios^44^. Higher water/fluid content is observed in GM tissues versus WM, and particularly in the caudate among sub-cortical regions explored here. This may be reflected here by lower signal fractions associated with the short T_2_* component observed in GM and the caudate in the current data (Fig. 3), and inversely higher values of Na_LF_ and the related metric EcF (Table 1, Fig. 4). Conversely, the white matter ROI in the centrum semiovale provides the highest short signal fraction (*f*) and the lowest estimates of Na_LF_ and EcF.

The extent and an-/isotropy of the mobility of nuclei also impacts relaxation, with the structure and cellular make-up and the constituents of intracellular and extracellular environments all potentially playing a role. The fluid environment in the interstitium between brain cells differs from the environment of CSF in the ventricles due to the presence of the negatively-charged long-chain molecules of the extracellular matrix^36,45^. The extracellular matrix regulates the ionic environment necessary for cellular functions such as synaptic transmission and action potentials, interacts with diffusion through extracellular spaces via viscous drag and the interaction with positive charged ion species such as ^23^Na, and varies in composition and density across brain structures^3,36,45^. The properties of the extracellular space and matrix are in turn influenced by cellular populations of neurons and glia, in terms of the distribution of different subtypes and the manner in which they constrain the structure of their environment via the formation of sheets, extension of processes and size alterations influencing the volume of intra-/extracellular spaces^36^.

Estimates from histology, DNA extraction and immunocytochemistry suggest that the human brain contains ~100 billion neurons and an equivalent number of non-neuronal cells including glia (oligodendrocytes > astrocytes > microglia)^46^ and a small proportion of endothelial cells, leading to an approximate overall glia-to-neuron ratio (GNR) of 1:1^47^. At the same time, GNRs vary widely between brain regions with extremes including the cerebellum (0.23) due to its small, densely packed neurons, while reaching 11.35 in other regions of the brain including brainstem, diencephalon and striatum (i.e. many of the subregions considered here)^47–50^. These variations are thought not to reflect differences in glia density, which varies much less between structures, but rather with increasing neuronal density that is itself due to decreasing neuronal cell size (including soma and dendritic and axonal arborisations)^47,49^. 500-fold variation in average neuronal cell masses are observed across structures and species versus 1.4-fold for glia^48^. The above evidence suggests that while the WM represents a restrictive environment determined mainly by the anisotropy^51^ of axons and small-bodied^46^ myelin-supporting oligodendrocytes, the GM represents a wider variety of cellular environments especially given the range of neuronal sizes. This is a possible explanation for the variation in relaxation times, associated signal fractions and derived metrics observed here. Similarly, these factors may contribute to the variation between regions (Fig. 4) to the extent that they reflect proportions of white and grey matter. It is notable that T_2_*_short_ and T_2_*_long,_ as well as Na_LF_ tend to be lowest in axon/myelin-rich structures. This is most evident across metrics in the pallidum and pons, but also to some extent in centrum semiovale, cerebellar white matter and the corpus callosum. It should be recognized that variability across metrics among these regions suggests additional factors at work as well.

Considering concordance of our results and the available relevant literature, in addition to recent insights into tissue composition, this raises the prospect of multi-echo ^23^Na-MRI’s sensitivity to microstructural properties of imaged tissues. Future work, including additional measures of microstructure, such as via the measurement of the diffusion properties of ^1^H or ^23^Na itself^14,15,18,52^, could confirm and expand the current results, and speak to limitations of the current approach. For example, despite the measures taken to avoid partial volume effects, as is the case for all ^23^Na-MRI there is a remaining possibility of an unknown bias due to the influence of higher concentrations of ^23^Na in extracellular and cerebrospinal fluid, and the possibility of differences between tissue types in terms of this bias. Further gains in spatial resolution could further ameliorate this issue^3^, as well as the application of partial volume correction methods originally applied in positron emission tomography and recently adapted to ^23^Na-MRI^27,41^. Given the duration of the scan required to characterise the decay curve, there is the possibility of artefacts arising from subject motion. Measures such as the use of foam supports to support subjects’ heads and constrain movement were employed; however optimisation of the sequence to shorten overall acquisition times should be pursued. In any case, this will be required for clinically feasible scan times.

In summary, we provide evidence that in the physiological state multi-echo ^23^Na-MRI depicts variation in T_2_* relaxation between major distinctions in tissue structure – GM and WM – as well as between diverse brain regions of differing composition and structure. This includes variations in decay times, including the T_2_*_short_ component. Additionally, quantification of ^23^Na concentrations apparent at short and long TEs was evaluated via a quantitative multi-echo approach for the first time. This provides a foundation for the examination of the alteration of these properties in pathophysiological populations, as a means of shedding light on both disease processes and the mechanisms underlying normal ionic homeostatic mechanisms as imaged by ^23^Na-MRI.

## Methods

### Subjects

14 healthy subjects with no history of neurological or psychiatric illness provided informed consent and were enrolled in the protocol, as approved by the local Ethics Committee (Comité de Protection des Personnes sud Méditerranée 1). All methods were performed in accordance with the relevant guidelines and regulations. Due to excessive movement in one subject, the final sample consisted of n = 13 subjects (5 female) (mean age: 23.9 ± 3.6 (s.d.) years, range: 20–32).

### MRI acquisition

Data were acquired on a whole-body 7 T Magnetom MR system (Siemens, Erlangen, Germany). ^23^Na-MRI was acquired using a dual-tuned ^23^Na/^1^H QED birdcage coil and a multi-echo density adapted 3D projection reconstruction pulse sequence (TR = 120 ms, 10000 spokes, 384 radial samples per spoke, radial fraction p of 0.32, 500 us hard RF pulse, bandwidth 200 Hz/pixel, 3.5 mm nominal isotropic resolution, 8 echoes, acquisition time = 20 min). To ensure a sufficient number and distribution of TEs, especially for measuring T_2_*_short_, while taking into account the 5 ms readout of the sequence, we applied the sequence three times in one exam in order to obtain 24 TEs ranging from 0.3 ms to 100 ms (Run 1: 0.3–6.3–13–19–25–31–54–89 ms; Run 2: 0.8–7–12–17–23–35–45–80 ms; Run 3: 2.3–9.7–15–21–28–40–66–100 ms). The three first subjects underwent an exam of 23 TEs across the same range (Run 1: 0.3–6.3–13–19–25–31–50–100 ms; Run 2: 2–7–12–17–23–40–45–80 ms; Run 3: 3–9–15–22–28–34–70 ms). Six cylinder tubes filled with 2% agar gel doped with a range of sodium concentrations (10–75 mM) were arrayed in the field of view in front of the subject’s head as external references for quantitative assessment of brain sodium concentration. Foam cushions were used to immobilize and support the head as well as the external references.

^1^H-MRI was performed during the same session as ^23^Na-MRI after a short pause to permit the changing of head coils. A high-resolution proton MRI 3D-MP2RAGE (TR = 5000 ms/TE = 3 ms/TI1 = 900 ms/TI2 = 2750 ms, 256 slices, 0.6 mm isotropic resolution, acquisition time = 10 min) was obtained using a 32-element (32Rx/1Tx) ^1^H head coil (Siemens).

### Pre-processing

Sodium images were reconstructed offline in Matlab (Mathworks, Natick, MA) using a Kaiser-Bessel gridding method^29^ leading to a full 3D data set for each individual, at each echo time (See Fig. 1 for an example). ^23^Na images from all TEs were realigned to the shortest TE (0.3 ms) for each individual. 3D ^23^Na images from the shortest TE were then realigned to high resolution 3D-MP2RAGE images obtained from the same individual and the resulting spatial transform was applied to the remaining ^23^Na images (SPM12; Wellcome Institute, London, UK). Subsequent analyses using ROIs (see below) were applied to these coregistered ^23^Na-MRI images in the native 3D-MP2RAGE space. Figure 2 provides an overview of the workflow applied to data.

Using SPM12, ^1^H images were segmented into probability maps of GM, WM and CSF to create masks to be applied to sodium images. A conservative probability threshold of 0.9 for tissue type segmentation was applied to GM and WM prior to the creation of masks. FSL (version 6.0, FMRIB, Oxford, UK) was used to define 8 regional sub-cortical ROIs using ^1^H images, thresholded GM and WM maps and ^23^Na images of the earliest (high SNR) TE (0.3 ms). Sodium images and thresholded tissue probability maps were projected onto ^1^H images to aid expert (S.G.) manual definition of ROIs for each individual. In addition to thresholding of tissue probability maps attempts to limit partial volume effects included the avoidance of tissue boundaries and evident areas of heightened signal due to the proximity of CSF spaces such as the ventricles during manual ROI definition (Fig. 2). In total, 10 ROIs were applied for each individual to derive mean ^23^Na signal (MARSBAR toolbox, SPM12) for each ROI for each echo time. ROIs correspond to the entire (thresholded) GM and WM segmentations, in addition to eight sub-cortical manual bilateral ROIs: thalamus, pallidum, putamen, caudate nucleus, corpus callosum, centrum semiovale, cerebellar white matter and pons.

### Biexponential model

Considering the design of the sodium sequence providing a maximum reduction of T1 weighting (TR = 120 ms, around four times longer than the T1 values^7^), determination of signal fractions and the time constants of short and long T2* components of signal decay in ^23^Na images can be modelled by a bi-exponential fitting procedure. For each ROI, we analysed the mean signal intensity across each of the 24 TEs and apply a biexponential model to the obtained curve (Fig. 2a) using the equation:

$$Signal=\sqrt{[{A}^{2}{(f\cdot {{\rm{e}}}^{\frac{-TE}{T{{2}^{\ast }}_{short}}}+(1-f)\cdot {{\rm{e}}}^{\frac{-TE}{T{{2}^{\ast }}_{long}}})}^{2}+\,Ri{c}^{2}]}$$

where A is an amplitude scaling term, *f* is the signal fraction associated with the short T_2_* component and Ric refers to a Rician noise-related scaling parameter as described in Gudbjartsson *et al*.^53^. The best-fit parameters were obtained from nonlinear least-squares (lsqnonlin) fitting. Model performance was measured by goodness of fit (r^2^). From the model we estimated a signal fraction for short (f) and long (1-f) T_2_* components, as well as estimating the time constants (T2*_short_ and T2*_long_). We calculated magnetization (M0) corresponding to the signal fraction estimated by the model in terms of the intercepts of the signal fraction components of the model, obtaining M0_SF_ = A·*f* and M0_LF_ = A·(1-*f*).

### Quantification: Na_SF_ & Na_LF_

As these signal fraction parameters are expressed in arbitrary units, we attempted to obtain quantitative values to access the same measurement scale across subject in order to explore variations in tissue types and regions, and permit literature comparisons. For each reference tube for each individual, we obtained the value of M0 and the time of transverse relaxation (T_2_*) for a given tube across the 24 TEs, through monoexponential fitting via MATLAB (R2012a, MathWorks) (Fig. 2b). We then model a linear relationship between obtained M0 and known concentrations across all tubes (Fig. 2c), which can be applied to parameters estimated from the biexponential model of *in vivo* data (M0_SF_ and M0_LF_, Fig. 2a). As such, the apparent concentrations equivalent to a given level of magnetization was obtained, yielding quantitative estimates (Na_SF_ and Na_LF_) for each brain ROI. Note that given that we have dynamic information in the form of the curve of signal intensity across TEs, we can hypothesize that at the longest TEs, the major signal contributors will be the slowest relaxing/most motile pools. Accordingly, the magnetization estimated from the intercept of the signal fraction associated with the long T_2_* component may be considered a good approximation of the sodium concentration of extracellular spaces. ‘Extracellular fraction’ (EcF) was obtained by the division of Na_LF_ by the commonly used value of 140 mM for extracellular Na concentration^7,15,54^. We also calculated the parameter total sodium concentration (TSC) as the sum of Na_SF_ and Na_LF_.

### Statistical analyses

Six dependant variables of interest were investigated via analysis in JMP v.9 (SAS Institute Inc., Cary, NC). These included T_2_*_short_ and T_2_*_long_ (ms) derived from the biexponential fitting procedure, the quantitative estimates Na_SF_ and Na_LF,_(mM), as well as TSC (mM) and EcF. An ANOVA was applied separately to the six dependant variables to investigate the factor of interest Tissue Type (2 levels: GM, WM), in addition to factors for Sex and Age. Additionally, an ANOVA was applied to the sub-cortical regional data, with the factor of interest Region (8 levels) as well as Sex and Age. Differences between the levels of factors of interest that were found to be significant were further investigated via non-parametric Steel-Dwass (p < 0.05, adjusted for multiple comparisons between levels) in a pairwise fashion. Based on the possibility of dependence between measures, Bonferroni correction was also applied across tests based on 6 dependant variables yielding a corrected α-level of *P* = 0.05/6 = 0.008.

### Data Availability

Generated datasets are available on reasonable request from the corresponding author.
